# Constituents of the Roots of *Dichapetalum pallidum* and Their Anti-Proliferative Activity

**DOI:** 10.3390/molecules22040532

**Published:** 2017-03-27

**Authors:** Dorcas Osei-Safo, Godwin Akpeko Dziwornu, Regina Appiah-Opong, Mary Anti Chama, Isaac Tuffour, Reiner Waibel, Richard Amewu, Ivan Addae-Mensah

**Affiliations:** 1 Chemistry Department, School of Physical and Mathematical Sciences, College of Basic and Applied Sciences, University of Ghana, P.O. Box LG 56, Legon, Accra, Ghana; dzwgod001@myuct.ac.za (G.A.D.); antichama@yahoo.com (M.A.C.); amewu@ug.edu.gh (R.A.); a-mensah@ug.edu.gh (I.A.-M.); 2 Department of Clinical Pathology, Noguchi Memorial Institute for Medical Research, College of Health Sciences, University of Ghana, P.O. Box LG 581, Legon, Accra, Ghana; ropong@gmail.com (R.A.-O.); ituffour@noguchi.ug.edu.gh (I.T.); 3 Department of Chemistry and Pharmacy, Friederich Alexander University of Erlangen-Nurnberg, Schuhstrasse 19, 91052 Erlangen, Germany; reiner.waibel@medchem.uni-erlangen.de

**Keywords:** *Dichapetalum pallidum*, 7-hydroxydichapetalin P, dichapetalin A, dichapetalin X, spiroketal, leukemia

## Abstract

As part of our search for bioactive compounds from the Dichapetalaceae, repeated chromatographic purification of the roots of a hitherto unexamined species, *Dichapetalum pallidum*, led to the isolation of the newly occurring 7-hydroxydichapetalin P (**1**) and the known dichapetalins A (**2**) and X (**3**). Also isolated were the known compounds friedelin-2,3-lactone (**4**), friedelan-3-one (**6**), friedelan-3β-ol (**7**) and pomolic (**8**), as well as the dipeptide aurantiamide acetate (**5**). The compounds were characterized by direct interpretation of their IR, 1D NMR and 2D NMR spectral data and by comparison of their physico-chemical data, including their chromatographic profiles, with the literature and authentic samples in our compound library for the genus *Dichapetalum*. The compounds were assayed for their anti-proliferative activities against the human T-lymphocytic leukemia (Jurkat), acute promyelocytic leukemia (HL-60) and T-lymphoblast-like leukemia (CEM) cell lines. Overall, dichapetalin X showed the strongest (3.14 μM) and broadest cytotoxic activities against all the leukemic cell lines tested, exhibiting even stronger activities than the standard compound, curcumin.

## 1. Introduction

*Dichapetalum* Thouars (Dichapetalaceae) is a rich source of triterpenoids and several species of the genus have produced a variety of this class of secondary metabolites. Although the genus was initially noted for being lethal to livestock and even humans, recent research has focused on the less toxic plant species and has led to the discovery of a novel class of phenylpyranotriterpenoid compounds, the dichapetalins. This unique class of triterpenoids in which a 2-phenylpyrano moiety is annellated to ring A of a 13,30-cyclodammarane-type triterpenoid is believed to be biogenetically derived from the condensation of the triterpenoid with a C_6_–C_2_ moiety, which is probably derived from the shikimic acid pathway. The main distinguishing feature of the dichapetalins is the nature of the side chain on the cyclopentane ring D which may comprise a lactone, methyl ester, spiroketal, lactol, acetyl and furan moieties. Originally known for their cytotoxic activity, dichapetalins with a spiroketal side chain have exhibited the most potent antineoplastic properties. Moreover, they have also been reported to have antioxidant, antihelminthic, antifeedant, nematicidal, antifungal, anti-HIV, nitric oxide– and acetylcholinesterase-inhibitory activities [1,2,3,4,5,6,7].

**Figure 1:**
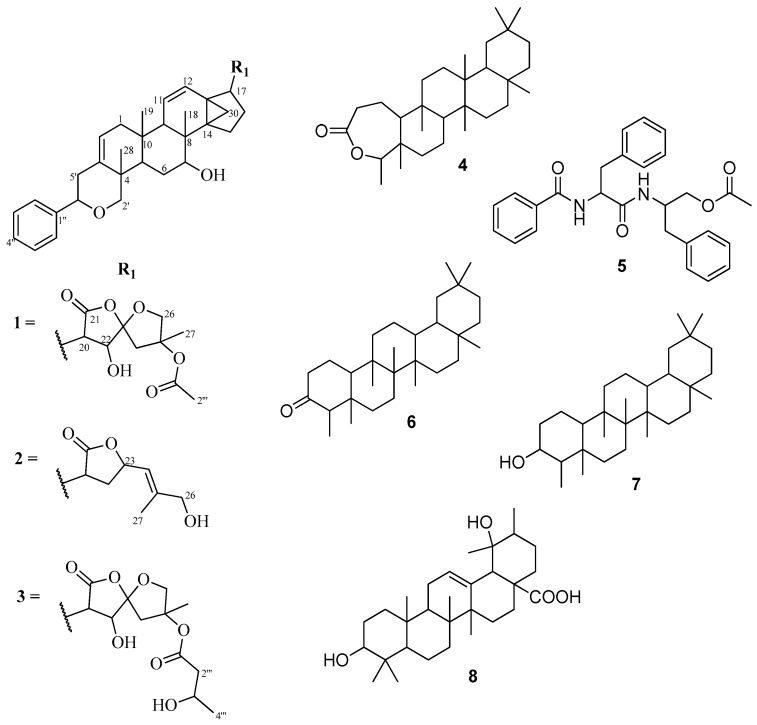
Structures of compounds **1**–**8**.

*Dichapetalum pallidum* (Oliv.) Engl. is distributed in west and central Africa. It has hitherto not been examined, neither for its chemical constituents nor for bioactivity. The main documented ethnobotanical applications are the use of the seeds as food in Togo and the leaves in the treatment of diarrhea [8]. As part of our on-going studies on the less common and less toxic species of the genus, in our search for new and more potent cytotoxic dichapetalins, we report the isolation and characterization of three dichapetalins from the roots of *D. pallidum*, namely 7-hydroxydichapetalin P (**1**), dichapetalin A (**2**) and the recently reported dichapetalin X (**3**) from *D. filicaule* [7]. Also isolated were friedelin-2,3-lactone (**4**), the dipeptide aurantiamide acetate (**5**), friedelan-3-one (**6**), friedelan-3β-ol (**7**) and pomolic acid (**8**) (Figure 1). Although dichapetalin P has been reported from *D*. *zenkeri* [5], its 7-hydroxy derivative (**1**) has hitherto not been reported as a naturally occurring compound. The dichapetalins exhibited better anti-proliferative activity against the human T-lymphocytic leukemia (Jurkat), acute promyelocytic leukemia (HL-60) and T-lymphoblast-like leukemia (CEM) cell lines than curcumin. The results further substantiate this class of dammarane-type triterpenoids as potential antitumor agents.

## 2. Results

### 2.1. Characterization of Compounds

Repeated silica gel column chromatographic separation of the ethyl acetate extract of the roots of *D. pallidum* afforded a total of seven triterpene derivatives and a dipeptide, aurantiamide acetate (**5**). Among the triterpenoids were dammarane-type 7-hydroxydichapetalin P (**1**), which is reported for the first time, dichapetalin A (**2**) and the recently reported dichapetalin X (**3**) from *D. filicaule* [7]. The remaining triterpenoids were the friedelane-type compounds friedelin-2,3-lactone (**4**), friedelan-3-one (**6**) and friedelan-3β-ol (**7**), and the ursane-type pomolic acid (**8**).

Compound **1** was obtained as a white crystalline solid. Five methyl protons were identified in the ^1^H-NMR spectrum within the range δ_H_ 0.92 to δ_H_ 2.03, the latter being assigned to an acetoxymethyl. The ^1^H-NMR spectrum exhibited signals due to five aromatic protons resonating at δ_H_ 7.24 (m, H-4″), δ_H_ 7.33 (m, H-3″, 5″) and δ_H_ 7.35 (m, H-2″, 6″). The ^13^C-NMR spectrum showed 38 signals. Two ester carbonyl functionalities were inferred by chemical shifts at δ_C_ 173.9 and δ_C_ 170.4 and supported by absorptions at 1780 and 1745 cm^−1^ in the IR spectrum. The intensity of the olefinic/aromatic signals at δ_C_ 125.8 and δ_C_ 128.4 appeared to be twice that of the other signals, indicating the presence of two sets of equivalent carbons. The HSQC data revealed 11 quaternary carbons interspersed within both the sp^2^- and sp^3^-hybridized regions, nine methylene, 13 methine and five methyl carbons. Thus, the expected total number of carbons present in **1** was 40.

**Figure 2:**
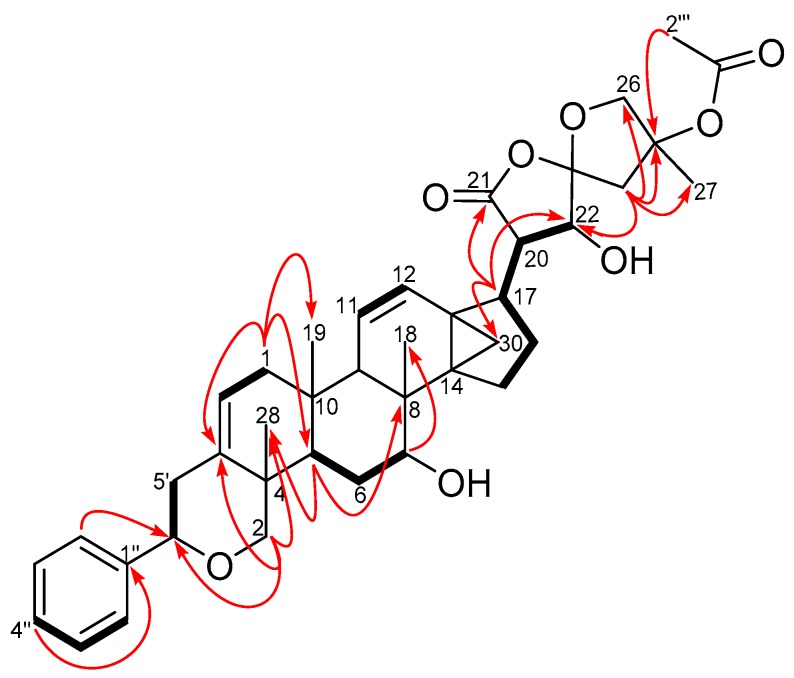
COSY (bold bonds) and some key HMBC (curved arrows) correlations of compound **1** (Supplementary Materials S4 and S6).

**Figure 3:**
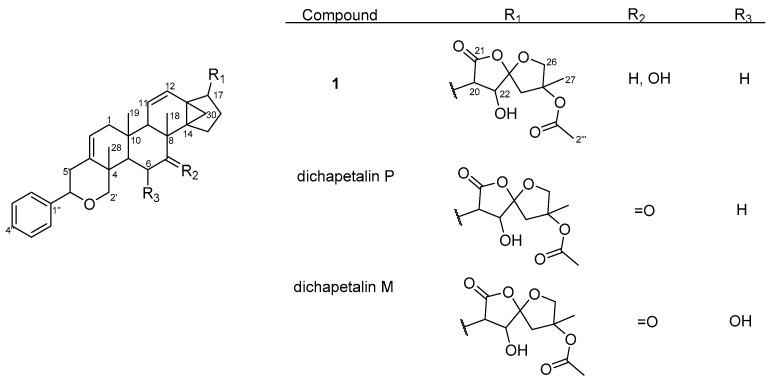
Structures of compound **1** and dichapetalins P and M.

Six ^1^H-^1^H COSY spin systems were observed (Figure 2), while some key HMBC correlations enabled the complete assignments of the ^1^H and ^13^C resonances of **1** which were consistent with previously accumulated data in our compound library of a dichapetalin carbon arrangement. For example, the 2-phenylpyrano moiety was corroborated from a mono-substituted phenyl ring comprising the four sp^2^ carbon signals C-1″ (δ_C_ 142.6), CH-2″, 6″ (δ_C_ 125.8, δ_H_ 7.35, m), CH-3″, 5″ (δ_C_ 128.4, δ_H_ 7.33, m) and CH-4″ (δ_C_ 127.5, δ_H_ 7.24, m), together with HMBC correlations from H-2″, 6″ to C-6′ (δ_C_ 81.8) and H-2′ to C-6′ and C-3 (δ_C_ 140.0) (Figure 2). The presence of the cyclopropane moiety, comprising C-13, -14 and -30, was supported by HMBC correlations from H-12 and H-17. The positions of the two isolated double bonds (C-2/C-3 and C-11/C-12), arising from four sp^2^ carbon signals at δ_C_ 117.8 (C-2), δ_C_ 124.1 (C-11), δ_C_ 129.1 (C-12), δ_C_ 140.0 (C-3) with corresponding olefinic proton multiplets at δ_H_ 5.42 (1H, dd, *J* = 1.8, 7.1 Hz, H-2), δ_H_ 5.46 (1H, dd, *J* = 2.6, 10.2 Hz, H-11), and δ_H_ 6.20 (1H, dd, *J* = 3.1, 10.2 Hz, H-12), were assigned based on HMBC correlations, for example from H_2_-1, H-2′ and H-9. In addition to an acetal at δ_C_ 111.3 (C-23), compound **1** revealed a spiroketal side chain at C-17 bearing an acetoxy group identical to those of dichapetalins M [4] and P [5] (Figure 3). However, the parent structure of the compound differed from those of both compounds, evident from an obvious absence of a C-7 ketocarbonyl in the ^13^C-NMR. This was replaced with a 7-OH group, supported by HSQC cross-peaks of C-7 (δ_C_ 72.3) to H-7 (δ_H_ 3.95), the ^1^H-^1^H COSY spin system involving H-5, H_2_-6 and H-7, and HMBC correlations to C-7 (δ_C_ 72.3) and C-8 (δ_C_ 36.4) from the methine proton H-5.

Thus, compound **1** was unequivocally identified as 7-hydroxydichapetalin P, the reduced form of dichapetalin P, which has hitherto not been reported as a naturally occurring dichapetalin. Moreover, **1** exhibited peaks in its IR spectrum and characteristic chromatographic profile with respect to its reaction with vanillin reagent as previously reported for the dichapetalins [5]. Based on the close similarity of its NMR data to previously reported congeners, compound **1** is most likely to share a relatively similar stereochemistry as its congeners.

Compounds **2** and **3** were identified as dichapetalins A and X, respectively, by comparison of their physico-chemical and spectroscopic data with published results [1,7] as well as by co-TLC with authentic samples in several solvent systems. Similarly, compounds **6**–**8** were also characterized respectively as friedelan-3-one, friedelan-3β-ol and pomolic acid on the basis of their physic-chemical and spectroscopic properties and comparison of the data with published data [7,9,10].

Compound **4** was isolated as a white crystalline solid. Eight methyl groups were confirmed by the following signals in the ^1^H-NMR spectrum: (δ_H_ 0.83, s, Me-25), (δ_H_ 0.89, s, Me-24), (δ_H_ 0.95, s, Me-26), (δ_H_ 0.99, s, Me-27), (δ_H_ 0.99, s, Me-30), (δ_H_ 1.00, s), (δ_H_ 1.17, s, Me-28) and (δ_H_ 1.20, d, *J* = 6.5 Hz, Me-23). The ^13^C-NMR indicated the presence of 30 carbons, the signal at δ_C_ 18.6 integrating for two carbons. Together with the DEPT and HSQC assignments, eight methyls, 11 methylenes, four methines and seven quaternary carbons including a carbonyl (δ_C_ 175.6) were identified, suggesting a triterpenoid skeleton. Further analyses of the NMR data showed a good comparison with the literature [11] for friedelin-2,3-lactone (**1**), (Figure 1). The HMBC correlations of C-4 (δ_C_ 84.9) to Me-23 (δ_H_ 1.20) and Me-24 (δ_H_ 0.89), and C-10 (δ_C_ 64.9) and C-3 (δ_C_ 175.6) to H-2a (δ_H_ 2.52) and H-2b (δ_H_ 2.63) supported the position of the lactone. Although the related compounds friedelan-3-one and friedelan-3β-ol are replete in the genus, this is the first report of the isolation of the lactone from the genus.

Compound **5** was obtained as a white amorphous solid. Its IR spectrum exhibited bands typical of an amide (3314 and 1661 cm^−1^), an acetoxy (1726 and 1261 cm^−1^), a monosubstituted phenyl (745 and 695 cm^−1^), and methyl, methylene and methine (2961, 2919, 2858 cm^−1^) groups, respectively. The presence of an acetoxymethyl was confirmed by a 3H singlet at δ_H_ 2.03 in the ^1^H-NMR spectrum. Signals for two amide NH groups were observed at δ_H_ 6.75 and δ_H_ 5.98. The ^13^C-NMR spectrum showed signals for three carbonyls, that is an acetoxy (δ_C_ 170.7), an aliphatic amide (δ_C_ 170.2) and an aromatic amide (δ_C_ 167.1), two benzylic methylenes and an oxymethylene signals at δ_C_ 37.8, δ_C_ 38.4 and δ_C_ 64.4, respectively, while two methines were found at δ_C_ 49.4 and δ_C_ 54.9, including the acetoxymethyl at δ_C_ 20.8. There were nine aromatic methines and three quaternary carbons within the range δ_C_ 126.7–δ_C_ 136.7, suggesting the presence of three mono-substituted phenyl rings. This was corroborated by 15 aromatic protons (δ_H_ 7.12–7.78) in the ^1^H-NMR spectrum. Comparison of the data with literature [12] led to the identification of compound **5** as the dipeptide aurantiamide acetate (Figure 1), previously isolated from red algae and several families of higher plants [13], but reported for the first time in the Dichapetalaceae.

### 2.2. Effect of Compounds ***1***–***5*** on Cell Proliferation of Leukemia Cancer Cell Lines

Human T-lymphocytic leukemia (Jurkat), acute promyelocytic leukemia (HL-60) and T-lymphoblast-like leukemia (CEM) cell lines were employed in the assay of the anti-proliferative activities of compounds **1**–**5**. Dichapetalins A and X were more active against CEM and HL-60 cells than 7-hydroxydichapetalin P and curcumin.

**Figure 4:**
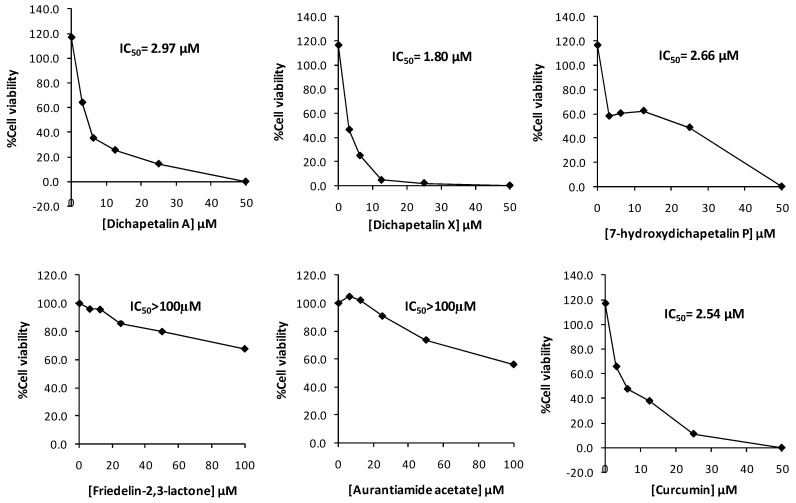
Cytotoxic effect of compounds **1**–**5** and curcumin on Jurkat cell viability.

In the initial screening of compounds **1**–**5** towards Jurkat cell lines, the dichapetalins strongly inhibited the growth of the Jurkat cells. The inhibitory activities (IC_50_ values) of 7-hydroxydichapetalin P (**1**), dichapetalin A (**2**) and dichapetalin X (**3**) were 2.66, 2.97 and 1.80 μM, respectively, while that for standard curcumin was 2.54 μM. On the other hand, friedelin-2,3-lactone (**4**) and aurantiamide acetate (**5**) showed rather weak inhibitory activities (IC_50_ > 100 µM) (Figure 4).

**Figure 5:**
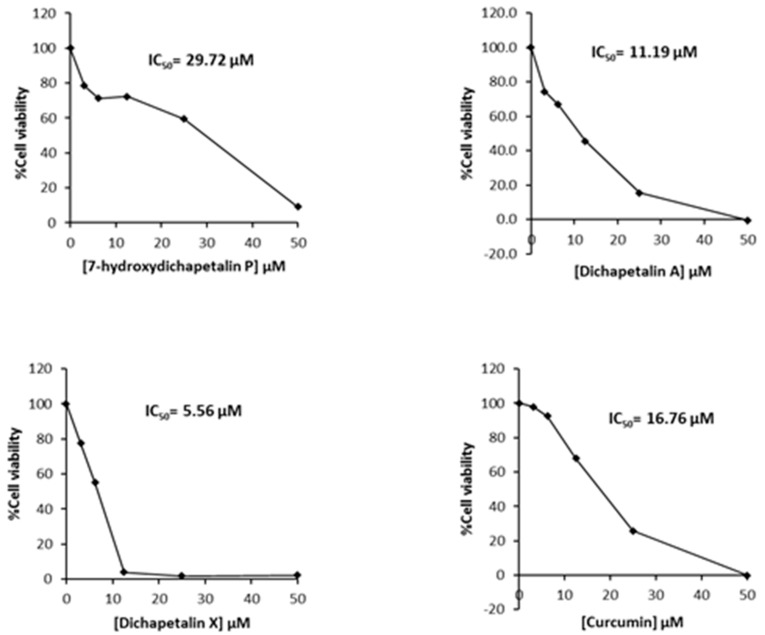
Cytotoxic effect of compounds **1**–**3** and curcumin on HL-60 cell viability.

**Figure 6:**
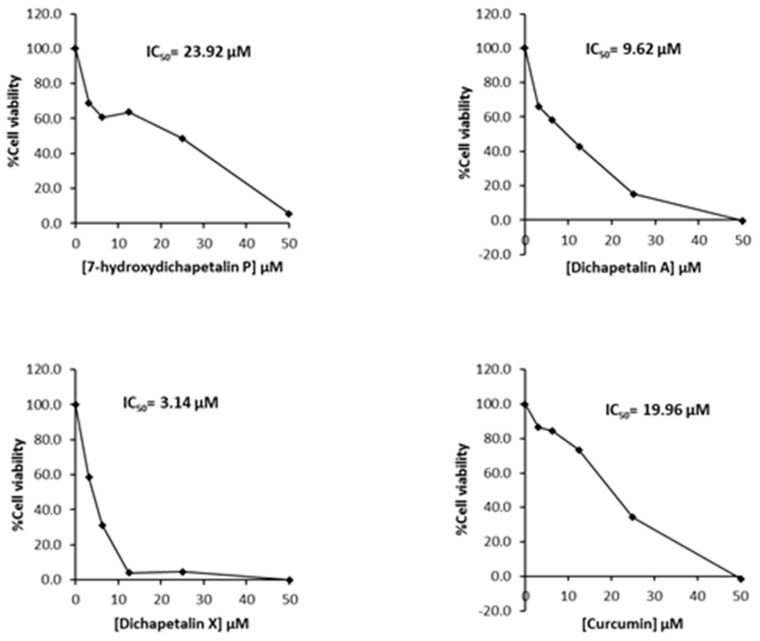
Cytotoxic effect of compounds **1**–**3** and curcumin on CEM cell viability.

Further testing of the dichapetalins in HL-60 and CEM cells revealed weaker inhibitory potency compared to those in Jurkat cells. In HL-60 cells, measured IC_50_ values were 29.72, 11.19 and 5.56 μM, respectively, for the dichapetalins and 16.76 μM for curcumin (Figure 5). This result confirms a previous assay in which the chemosensitivity of dichapetalin A on HL-60 was reported as 11.0 µM^5^. The CEM cells were slightly more sensitive to the dichapetalins than HL-60 cells, with IC_50_ values of 23.92, 9.62 and 3.14 μM, respectively, for 7-hydroxydichapetalin P, dichapetalin A and dichapetalin X, but more resistant to curcumin (IC_50_ = 19.96 μM) (Figure 6).

Overall, dichapetalin X (**3**) showed the strongest and broadest cytotoxic activities against all the leukemic cell lines tested, exhibiting even stronger activities than curcumin. Although 7-hydroxydichapetalin P (**1**) was in all cases a weaker inhibitor than curcumin, their IC_50_ values were comparable, indicating that the dichapetalins isolated in this study are cytotoxic to all the leukemia cell lines tested. This suggests that the compounds are potential anticancer agents, corroborating earlier studies that have indicated that the dichapetalins have anticancer properties [2,3,5,6]. So far, dichapetalins M and P, which contain an intact lactone in the side chain and a ketocarbonyl group at C-7, have exhibited the most potent anti-proliferative activities, with dichapetalin M exhibiting nanomolar cytotoxicity towards human colorectal carcinoma (HCT-116) [5]. These two structural features have therefore been implicated in the pharmacological action of this class of compounds. Dichapetalin X and 7-hydroxydichapetalin P have the intact lactone but lack the 7-carbonyl, while both moieties are absent in dichapetalin A. Thus, structurally, 7-hydroxydichapetalin P and dichapetalin P are identical, with the exception of the substituent at the 7 position. Dichapetalin M is 6-hydroxydichapetalin P. Dichapetalin X, on the other hand, has a different side chain where the acetoxy group in the dichapetalin P derivatives is replaced with a butyloxy substituent. These findings suggest the need to carry out further studies to confirm the proposed structure-activity relationship and to determine the mechanisms of action of this intriguing class of triterpenoids.

## 3. Conclusions

The identification of dichapetalins in *D. pallidum*, hitherto unexamined for its chemical constituents, is of chemotaxonomic significance. All the isolated dichapetalins exhibited micromolar anti-proliferative activity towards three leukemia cancer cell lines, namely the Jurkat, HL-60 and CEM cell lines. Dichapetalin X, recently isolated as a novel dichapetalin from *D. filicaule* with potential antihelminthic activity, but not assayed for its anti-proliferative activity, was the most potent cytotoxic agent against all the leukemic cell lines tested. It exhibited even stronger activity towards these cell lines than the standard compound used, curcumin. The presence of a spiroketal moiety in its side chain further supports the suggested relationship of this structural feature to the potent anti-proliferative activity in this class of compounds.

In order to establish the identity of the pharmacophores, it is important to investigate the mechanisms underlying the observed anti-proliferative properties of the dichapetalins and possibly incorporate these pharmacophores onto smaller scaffolds as a means of synthesizing simple but potent anticancer agents.

## 4. Materials and Methods

### 4.1. General Experimental Procedures

TLC was performed on aluminum foil slides pre-coated with silica gel (thickness 0.2 mm, type Kiesegel 60 F_254_ Merck (Merck KGaA, Darmstadt, Germany) using petroleum ether/Me_2_CO mixtures, petroleum ether/Me_2_CO/CHCl_3_ (7:5:5); petroleum ether/Et_2_O (3:7); petroleum ether/EtOAc (10:3); toluene/EtOH mixtures; CHCl_3_ (100%). Detection: I_2_ vapor, anisaldehyde spray reagent, and H_2_SO_4_ spray reagent. All chromatographic separations were carried out by column chromatography (CC) on Fluka silica gel 60 (Sigma-Aldrich, Buchs, Switzerland). Melting points (uncorrected) were determined on a Stuart Scientific Melting Point Apparatus (Cole-Parmer, Staffordshire, UK). IR spectra were recorded in KBr discs on a Shimadzu IR-408 spectrophotometer (Shimadzu, Burladingen, Germany). ^1^H-NMR was either run at 600 MHz on a Brüker Avance 600 Spectrophotometer (Bruker, Billerica, MA, USA) or at 500 MHz on a Brüker Ascend^TM^ 500 Spectrophotometer in CDCl_3_ with TMS as the internal standard.

### 4.2. Plant Material

Whole roots and stem of *Dichapetalum pallidum* were collected from Asenanyo Forest Reserve (Ashanti Region, Ghana) in July 2013 and identified by Mr. John Ntim-Gyakari, formerly of the Forestry Commission, Kumasi. A voucher specimen (DPA001) was deposited at the National Herbarium, Botany Department, University of Ghana, Legon.

### 4.3. Extraction and Isolation

The air-dried pulverized whole root of *D.* *pallidum* (1786 g) was extracted with EtOAc (4 L, 24 h) by soxhlet. After concentration under reduced pressure, the dark brown crude extract (38.7 g) was chromatographed on a silica gel column, eluting with petroleum ether/EtOAc (10:1 to 0:10, *v*/*v*) to obtain fractions F1–F5. Fraction F1 (2.14 g) was re-chromatographed with petroleum ether/EtOAc (15:0.5) to yield three oily subfractions, F11–F13. Subfraction F12 (942 mg) on trituration in petroleum ether followed by further CC with CHCl_3_/petroleum ether (3:1, *v*/*v*) gave friedelan-3-one (**6**) (240 mg), friedelan-3β-ol (**7**) (20 mg) and a mixture of the two compounds. Fraction F2 (4.54 g) yielded nine subfractions, F21–F29, when chromatographed with petroleum ether/EtOAc (5:1). Subfractions F23 to F25 (933 mg) were combined and triturated with petroleum ether to afford an impure solid (280 mg) which on further chromatography in CHCl_3_ afforded friedelin-2,3-lactone (**4**) (64 mg) and β-sitosterol + stigmasterol (20 mg). Subfraction F29, on trituration with petroleum ether/EtOAc (4:1) precipitated pomolic acid (**8**) (37 mg). Seven subfractions (F31 – F37) were obtained when fraction F3 (1.35 g) was further separated by CC eluting with petroleum ether/EtOAc (5:1). Subfractions F33 and F35 to F37 were separately triturated with Et_2_O to yield aurantiamide acetate (**5**) (80 mg), 7-hydroxydichapetalin P (**1**) (20 mg), dichapetalin A (**2**) (25 mg) and dichapetalin X (**3**) (130 mg). Fraction F4 (1.15 g) was subjected to CC to yield subfractions (F41 – F45) while F5 precipitated a solid, F5S1. No further analysis could be carried out on these due to paucity of material.

*7-Hydroxydichapetalin P* (**1**): White crystals (20 mg); m.p.: 249–251 °C; IR ν_max_ cm^−1^ (KBr): 3551, 3370 (OH hydrogen bonded), 2973, 2919 (methyl), 1782 (>C=O lactone), 1746 (>C=O ester), 1246, 1094, 947, 700 (C_6_H_5_ bending). ^1^H-NMR and ^13^C-NMR, see Table 1.

*Dichapetalin A* (**2**): White crystals (25 mg); m.p.: 224–226 °C (Lit.: 228–230 °C, [4]); IR ν_max_ cm^−1^ (KBr): 3573, 3536, 3360, 2957, 2931, 1745, 1602, 1449, 1191, 1071, 1050, 766, 701 (Lit.: 3573, 3535, 3369, 2957, 2932, 1713, 1654, 1449, 1191, 1071, 1050, 767, 701 [7]); Co-TLC with authentic sample in petroleum ether/CHCl_3_/Me_2_CO (7:5:5), *R*_f_ = 0.82; petroleum ether/EtOAc (10:3), *R*_f_ = 0.46; CHCl_3_ (100%); *R*_f_ = 0.58, anisaldehyde: purple. ^1^H-NMR and ^13^C-NMR, see Table 1.

*Dichapetalin X* (**3**): White crystals (130 mg); m.p.: 244–246 °C (Lit.: 246–248 °C, [7]); IR ν_max_ cm^−1^ (KBr): 3549, 3367, 2973, 2921, 1784, 1743, 1167, 699 (Lit.: 3577, 3368, 2973, 2921, 1782, 1743, 1602, 699 [7]); Co-TLC with authentic sample in petroleum ether/CHCl_3_/Me_2_CO (7:5:5), *R*_f_ = 0.82; petroleum ether/EtOAc (10:3), *R*_f_ = 0.46; CHCl_3_ (100%); *R*_f_ = 0.58, anisaldehyde: purple. ^1^H-NMR and ^13^C-NMR, see Table 1. The values are agreeable to those reported by Chama et al. [7].

*Friedelin-2,3-lactone* (**4**): White crystals (64 mg); m.p.: 285–288 °C (Lit.: 288–290 °C, [11]); ^1^H-NMR (500 MHz, CDCl_3_) δ_H_: 0.83 (3H, s, Me-25), 0.89 (3H, s, Me-24), 0.95 (3H,s, Me-26), 0.99 (6H, s, Me-27, Me-30), 1.00 (3H, s, Me-29), 1.17 (3H, s, Me-28), 1.20 (3H,d, Me-23), 1.93 (1H, m), 2.52 (1H,td, H-2), 2.63 (1H,ddd, H-2), 4.22 (1H,q, H4). ^13^C-NMR (150 MHz, CDCl_3_) δ_C_: 18.6 (CH_2_, C-1), 34.4 (CH_2_, C-2), 175.6 (C, C-3), 84.9 (CH, C-4), 40.8 (C, C-5), 38.4 (CH_2_, C-6), 18.0 (CH_2_, C-7), 52.7 (C, C-8), 38.2 (C, C-9), 63.9 (CH, C-10), 35.3 (CH_2_, C-11), 30.6 (CH_2_, C-12), 39.3 (C, C-13), 38.4 (C, C-14), 32.4 (CH_2_, C-15), 35.4 (CH_2_, C-16), 30.0 (C-17), 42.7 (CH, C-18), 36.0 (CH_2_, C-19), 28.2 (C, C-20), 32.7 (CH_2_, C-21), 39.2 (CH_2_, C-22), 13.5 (CH_3_, C-23), 16.2 (CH_3_, C-24), 17.9 (CH_3_, C-25), 18.6 (CH_3_, C-26), 20.2 (CH_3_, C-27), 32.1 (CH_3_, C-28), 31.8 (CH_3_, C-29), 35.0 (CH_3_, C-30).

*Aurantiamide acetate* (**5**): White powdery/amorphous, (80 mg); m.p.: 177–180 °C (Lit.: 184 °C, [13]). IR in KBr; ν_max_ cm^−1^: 3314 (NH), 1661 (-CON), 1726 and 1261 (OAc), 745 and 695 (monosubstituted phenyl), methyl (2961), methylene (2919) methine (2858). ^1^H-NMR (600 MHz, CDCl_3_) δ_H_: 7.07–7.73 (15H, aromatic protons), 6.75 (1H, d, *J* = 7.7 Hz, N-H), 5.98 (1H, d, *J* = 8.4 Hz, N-H), 4.78 (1H, dd, *J* = 5.6 Hz, H-7), 4.35 (1H, m, H-4), 3.93 (1H, dd, *J* = 11.9, 4.9 Hz, H-3b), 3.83 (1H, dd, *J* = 11.9, 4.2 Hz, H-3a), 3.22 (1H, dd, *J* = 13.3, 6.3 Hz, H-10b), 3.07 (1H, dd, *J* = 13.3, 8.4 Hz, H-10a), 2.75 (2H, m, H-11), 2.03 (3H, s, AcCH_3_). ^13^C-NMR (600 MHz CDCl_3_) δ_C_: 170.7 (C-2, ester), 170.2 (C, C-6, aliphatic amide), 167.1 (C, C-9, aromatic amide), 136.7 (C, C-1′′′), 136.6 (C, C-1z), 133.7 (C, C-1′), 131.9 (CH, C-4′), 129.3 (CH, C-4″), 129.1 (CH, C-4′′′), 128.7 (CH, C-2′′′/C-6′′′), 128.6 (CH, C-2′/C-6′), 128.6 (CH, C-3′/C-5′), 127.1 (CH, C-3″/C-5″), 127.0 (CH, C-2″/C-6″), 126.7 (CH, C-3′′′/C-5′′′), 64.6 (CH_2_, C-3), 54.9 (CH, C-7), 49.4 (CH, C-4), 38.4 (CH_2_, C-11), 37.4, (CH_2_, C-10), 20.8 (CH_3_, C-1).

*Friedelan-3-one* (**6**). White needles (240 mg); m.p.: 255–257 °C (from EtOH) (Lit.: 258–259 °C, [9]); IR ν_max_ cm^−1^ (KBr): 2972, 2927, 2870, 1716, 1462, 1389 (Lit.: 2971, 2926, 2869, 1715, 1463, 1389 [9]). Co-TLC with authentic sample in petroleum ether/Me_2_CO (12:0:5), R_f_ = 0.80; toluene/EtOH (14:0.1), *R*_f_ = 0.94; CHCl_3_ (100%); *R*_f_ = 0.83, anisaldehyde: yellow, darkens with time.

*Friedelan-3β-ol* (**7**). White crystals (20 mg); m.p.: 276–278 °C (from EtOH) (Lit.: 274–276 °C [7]); IR ν_max_ cm^−1^ (KBr) 3621, 3480, 2915, 1451, 1385 (Lit.: 3619, 3474, 2911, 2870, 1448, 1385, [7]); Co-TLC with authentic sample in petroleum ether/Me_2_CO (12:0:5), *R*_f_ = 0.51; toluene/EtOH (14:0.1), *R*_f_ = 0.76; CHCl_3_ (100%); *R*_f_ = 0.67, anisaldehyde: purple.

*Pomolic acid* (**8**): White powder (37 mg); m.p.: 289–292 °C (from absolute EtOH) (Lit.: 286–289 °C [10]); Anisaldehyde: purple; IR ν_max_ cm^−1^ (KBr) 3569, 3413, 2875, 1687, 1462, 1388, 1269, 1073 (Lit.: 3569, 3413, 2875, 1687, 1461 [7]); Co-TLC with authentic sample in petroleum ether/Me_2_CO (10:3), *R*_f_ = 0.48; petroleum ether/Et_2_O (3:7), *R*_f_ = 0.78; toluene/EtOH (8:1), *R*_f_ = 0.47, anisaldehyde: purple.

### 4.4. Cytotoxicity Assay

The cytotoxic activities of the compounds towards Jurkat, HL-60, CEM and MCF-7 cancer cell lines were determined using the MTT assay [14]. The cells were cultured in RPMI supplemented with 10% fetal bovine serum (FBS) and 1% penicillin-streptomycin. The cells were then seeded in a 96-well plate at 1 × 10^4^ cells/well and treated with varying concentrations of the compounds (0–100 µg/mL) and maintained in a humidified incubator at 37 °C in the presence of 5% CO_2_ incubated for 72 h. Twenty microliters of 2.5 mg/mL MTT solution in phosphate buffered saline (PBS) was added to each well and incubation was continued for 4 h. Acidified isopropanol containing 1% Triton-X was added to each well, and subsequently the plates were kept in the dark at room temperature to dissolve any formazan crystals formed. Absorbance readings of wells were measured at 570 nm with a microplate reader (Tecan Infinite M200, Grödig, Austria). All experiments were performed in triplicate.

## Figures and Tables

**Table 1:** NMR spectroscopic data for 7-hydroxydichapetalin P (**1**), dichapetalins A (**2**) and X (**3**).

No.	1 (in CDCl_3_, 600 MHz)		2 (in CDCl_3_, 600 MHz)		3 (in CDCl_3_, 500 MHz)	
	δ_c_, Type	δ_H_ (*J*/Hz)	δ_c_, Type	δ_H_ (*J*/Hz)	δ_c_, Type	δ_H_ (*J*/Hz)
**1a**		1.68, m		1.66, m		1.69, m
**1b**	40.1, CH_2_	2.03, dd (7.1, 16.1)	40.1, CH_2_	2.10, dd (7.0, 16.2)	40.1, CH_2_	2.11, dd (7.1, 16.2)
**2**	117.8, CH	5.42, d (7.1)	117.7, CH	5.40, d (6.9)	117.8, CH	5.43, ddd (1.8, 1.8, 7.1)
**3**	140.0, C		140.1, C		140.0, C	
**4**	38.3, C		38.3, C		38.3, C	
**5**	43.7, CH	2.01, m	43.8, CH	2.00, m	43.8, CH	2.04, m
**6a**		1.62, ddd (2.4, 13.8, 13.8)		1.62, m		1.67, m
**6b**	24.1, CH_2_	1.85, ddd (3.0, 3.0, 13.8)	24.2, CH_2_	1.85, dd (10.5, 12.7)	24.2, CH_2_	1.86, ddd (3.0, 3.0, 13.8)
**7**	72.3, CH	3.95, dd (2.4, 3.0)	72.3, CH	3.94, m	72.3, CH	3.97, dd (2.4, 3.0)
**8**	36.4, C		36.4, C		36.4, C	
**9**	45.7, CH	2.01, m	45.7, CH	1.98, m	45.7, CH	2.02, m
**10**	35.7, C		35.2, C		35.7, C	
**11**	124.1, CH	5.46, dd (2.5, 10.2)	124.1, CH	5.45, dd (2.1, 10.0)	124.0, CH	5.48, dd (2.6, 10.2)
**12**	129.1, CH	6.20, dd (3.1, 10.2)	129.0, CH	6.14, dd (2.9, 10.0)	129.1, CH	6.21, dd (3.1, 10.2)
**13**	29.9, C		30.1, C		30.0, C	
**14**	36.2, C		36.2, C		36.3, C	
**15a**		1.73, m				
**15b**	24.8, CH_2_	2.05, m	24.9, CH_2_	2.05, d (12.2)	24.8, CH_2_	2.08, m
**16a**		1.27, m				
**16b**	23.2, CH_2_	1.78, ddd (9.6, 15.6, 19.2)	22.8, CH_2_	1.12, m	23.2, CH_2_	1.78, ddd (9.6, 15.6, 19.2)
**17**	40.3, CH	2.63, m	41.0, CH	2.62, m	40.3, CH	2.64, m
**18**	17.5, CH_3_	0.92, s	17.4, CH_3_	0.90, s	17.5, CH_3_	0.92, s
**19**	18.2, CH_3_	1.08, s	18.1, CH_3_	1.08, s	18.2, CH_3_	1.11, s
**20**	47.1, CH	3.01, dd (5.1, 10.0)	42.1, CH	3.08, ddd (4.9, 8.3, 12.9)	47.0, CH	3.05, dd (5.1, 10.0)
**21**	173.9, C		178.2, C		173.9, C	
**22**	71.9, CH	4.18, t (9.6)	31.4, CH_2_	2.37, m	72.1, CH	4.19, d (9.5)
**23**	111.3, C		75.1, CH	5.12, m	111.4, C	
**24a**		2.51, d (15.1)				2.53, d (15.2)
**24b**	45.7, CH_2_	2.84, d (15.1)	122.0, CH	5.52, dd (8.3, 1.0)	45.8, CH_2_	2.91, d (15.2)
**25**	85.0, C		141.7, C		86.0, C	
**26a**		4.09, d (10.2)				4.09, d (10.2)
**26b**	78.9, CH_2_	4.32, d (10.2)	67.2, CH_2_	4.05, s	78.3, CH_2_	4.37, d (10.2)
**27**	22.0, CH_3_	1.70, s	14.1, CH_3_	1.74, s	21.6, CH_3_	1.74, s
**28**	23.8, CH_3_	1.33, s	23.8, CH_3_	1.32, s	23.8, CH_3_	1.36, s
**30a**		0.93, d (5.3)		0.78, d (5.2)		0.96, d (5.3)
**30b**	15.4, CH_2_	1.29, m	15.0, CH_2_	1.18, d (4.6)	15.5, CH_2_	
**2′a**		3.62, d (10.5)		3.59, d (10.6)		3.63, d (10.5)
**2′b**	72.5, CH_2_	3.80, d (10.5)	72.5, CH_2_	3.76, d (10.6)	72.5, CH_2_	3.80, d (10.5)
**5′a**		2.20, dd		2.19, dd (2.4, 13.4)		2.23, dd (2.7, 13.7)
**5′b**	40.7, CH_2_	2.63, m	40.7, CH_2_	2.62, m	40.8, CH_2_	
**6′**	81.8, CH	4.28, dd (2.5, 9.1)	81.8, CH	4.26, dd (2.3, 11.6)	81.8, CH	4.29, dd (3.6, 11.6)
**1″**	142.6, C		142.6, C		142.6, C	
**2″,6′′**	125.8, CH	7.35, m	125.8, CH	7.37, m	125.8, CH	7.36, m
**3″,5″**	128.4, CH	7.33, m	128.3, CH	7.33, m	128.4, CH	7.40, m
**4″**	127.5, CH	7.24, m	127.5, CH	7.25, m	127.5, CH	7.28, m
**1′′′**	170.4, C				172.1, C	
**2′′′a**	21.8, CH_3_	2.03, s				2.40, dd (9.2, 16.2)
**2′′′b**					43.7, CH_2_	2.47, dd (3.6, 16.2)
**3′′′**					64.6, CH	4.20, m
**4′′′**					22.7, CH_3_	1.26, d (6.1)

## Supplementary Materials

Supplementary materials are available online. Scheme S1: Isolation of compounds **1**–**8**, Figure S1: The structures of compounds **1**–**8**, Figures S2–S11: Spectroscopic data for compounds **1**–**3**, Tables S1–S3: Biodata for compounds **1**–**3**.
