# Supplementary material for: Constituents of the Roots of Dichapetalum pallidum and Their Anti-Proliferative Activity
Source: Molecules. 2017 Mar 27;22(4):532. doi: 10.3390/molecules22040532 (PMC6154325; doi:10.3390/molecules22040532)
Supplement: Supplementary file 1 [file molecules-22-00532-s001.pdf]

Supplementary Data: Constituents of the roots of *Dichapetalum pallidum* and their anti-proliferative activity

Dorcas Osei-Safo, Godwin Akpeko Dziwornu, Regina Appiah-Opong, Mary Anti Chama, Isaac Tuffour, Reiner Waibel, Richard Amewu and Ivan Addae-Mensah

Table of Contents:

Scheme S1. Isolation of compounds **1 – 8**

Figure S1. The structures of compounds **1 – 8**

Figure S2. ^{1}H-NMR spectrum (600MHz, $CDCl_3$, 303K) of compound **1**

Figure S3. ^{13}C-NMR spectrum (150MHz, $CDCl_3$, 303K) of compound **1**

Figure S4. COSY NMR spectrum (600MHz, $CDCl_3$, 303K) of compound **1**

Figure S5. HSQC NMR spectrum (600MHz, $CDCl_3$, 303K) of compound **1**

Figure S6. HMBC NMR spectrum (600MHz, $CDCl_3$, 303K) of compound **1**

Figure S7. ^{1}H NMR spectrum (600MHz, $CDCl_3$, 303K) of compound **2**

Figure S8. ^{13}C NMR spectrum (150MHz, $CDCl_3$, 303K) of compound **2**

Figure S9. ^{1}H NMR spectrum (600MHz, $CDCl_3$, 303K) of compound **3**

Figure S10. ^{13}C NMR spectrum (150MHz, $CDCl_3$, 303K) of compound 3

Figure S11. IR spectrum of compound **1**

Table S1. Jurkat cell viability assay data for compounds **1 - 3**

Table S2. HL-60 cell viability assay data for compounds **1 – 3**

Table S3. CEM cell viability assay data for compounds **1 – 3**

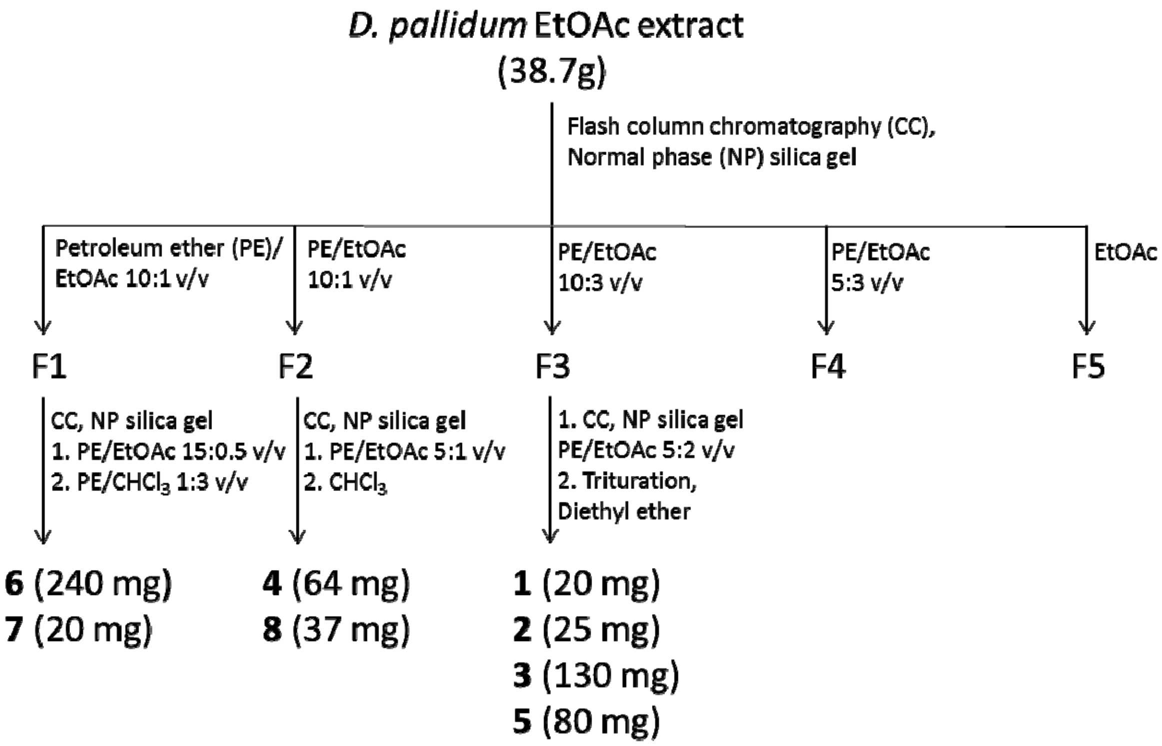

Scheme S1. Isolation of compounds **1** – **8**

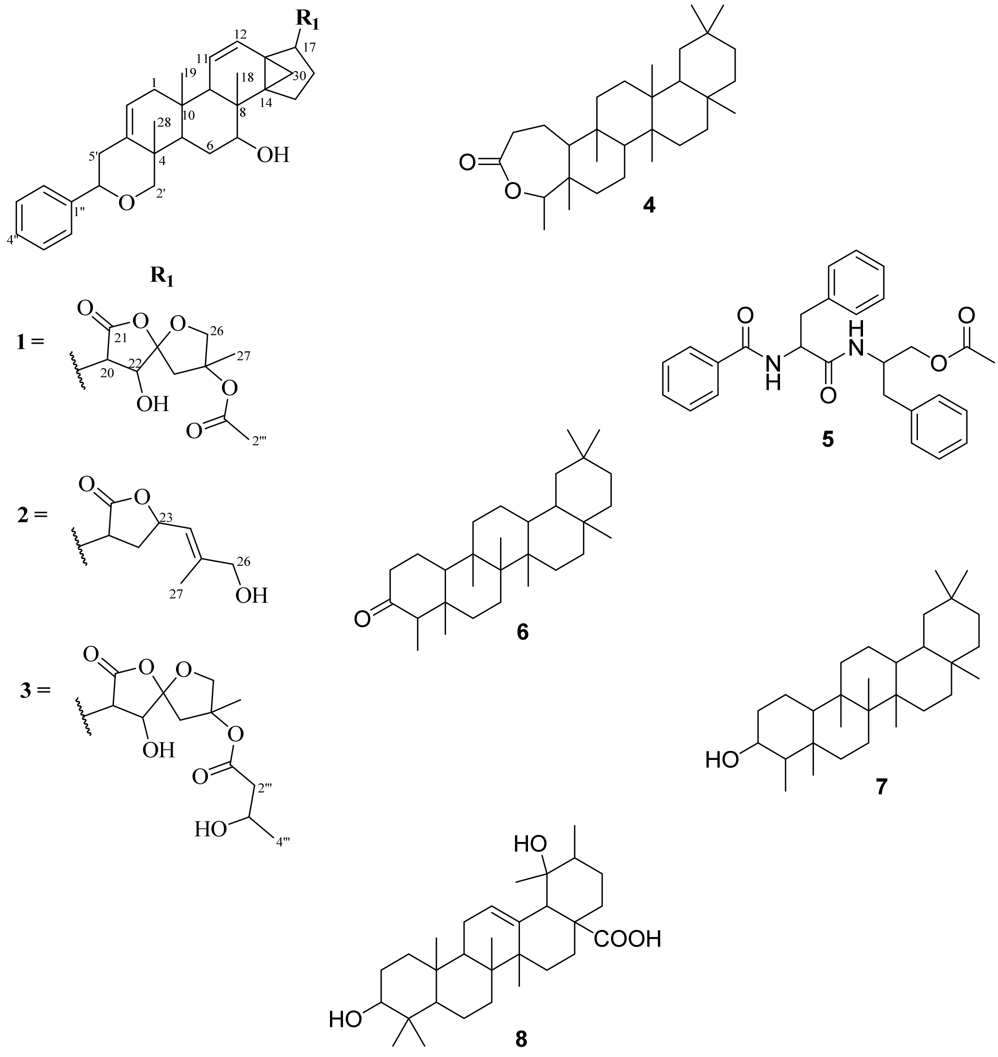

Figure S1. The structures of compounds **1** – **8**

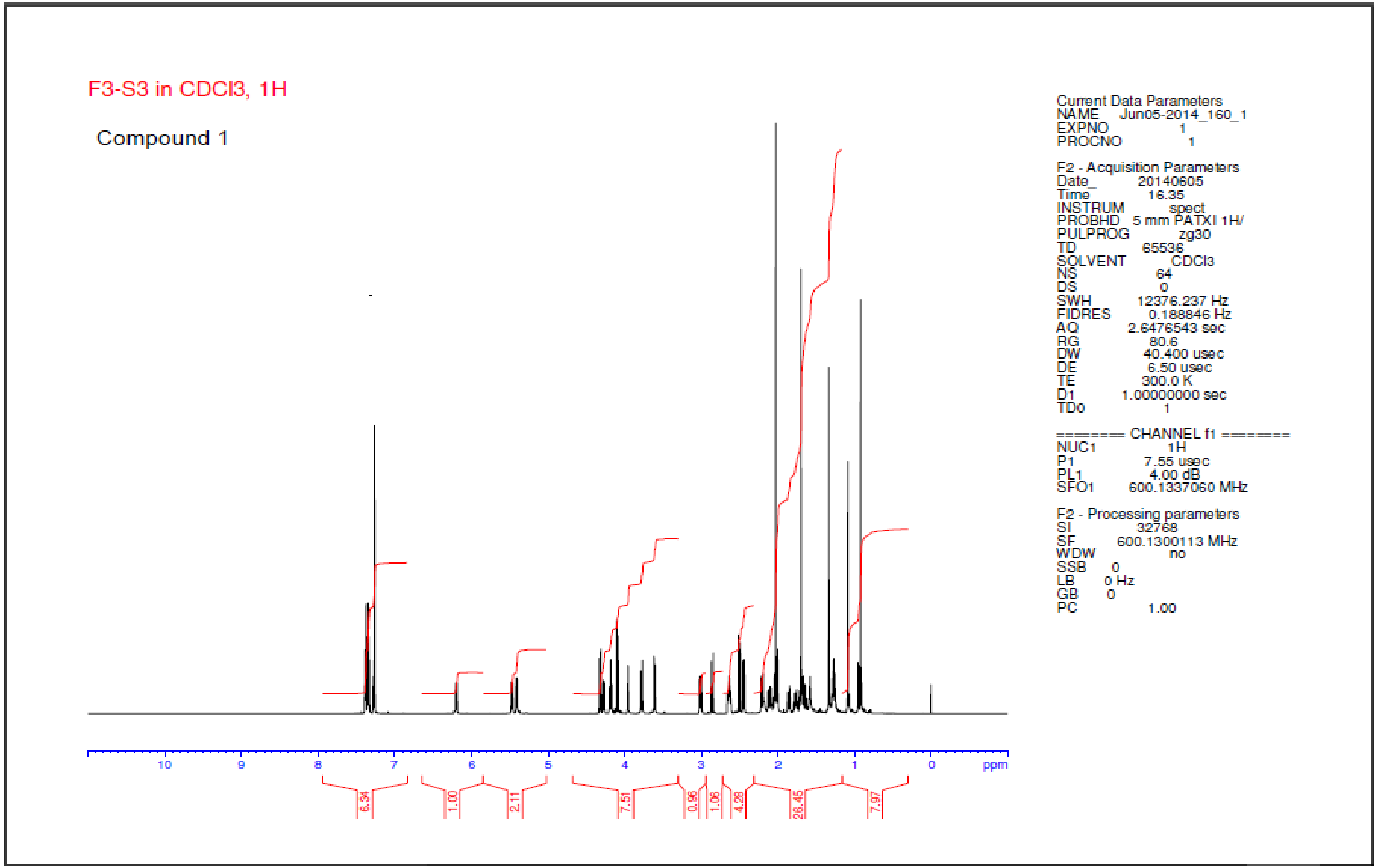

Figure S2. ^{1}H-NMR spectrum (600MHz, $CDCl_3$, 303K) of compound 1

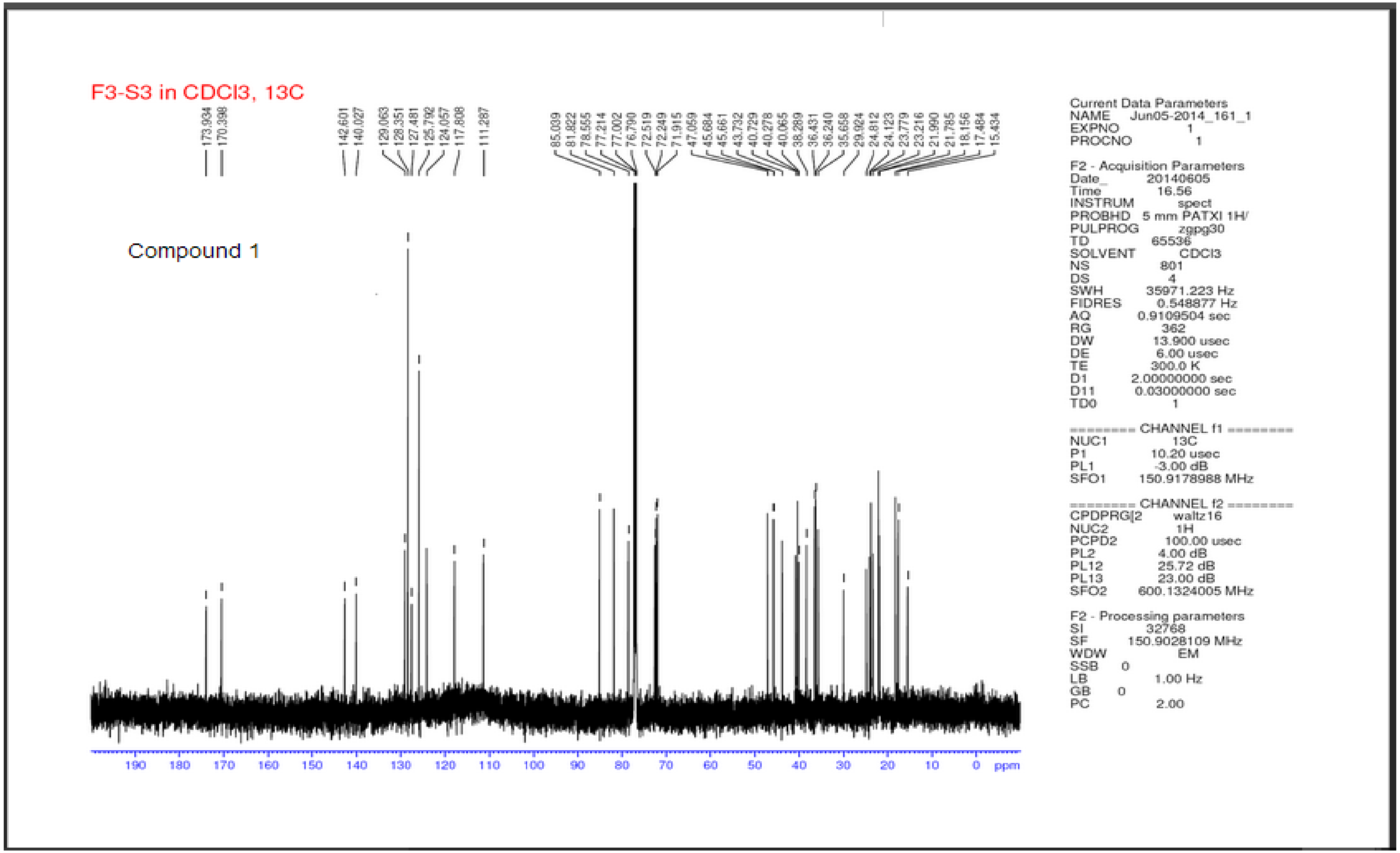

Figure S3. ^{13}C-NMR spectrum (150MHz, $CDCl_3$, 303K) of compound 1

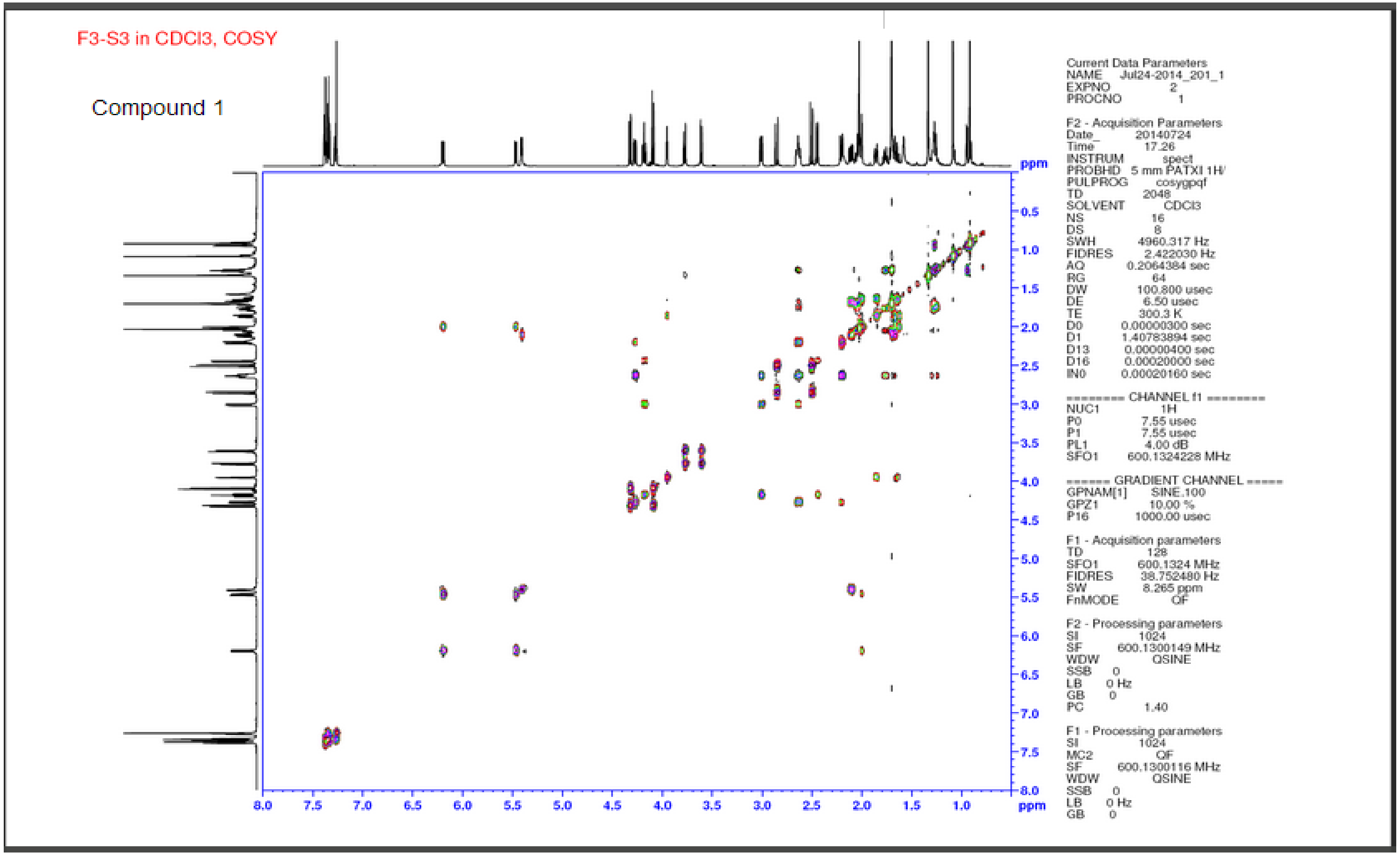

Figure S4. COSY spectrum (600MHz, $CDCl_3$, 303K) of compound 1

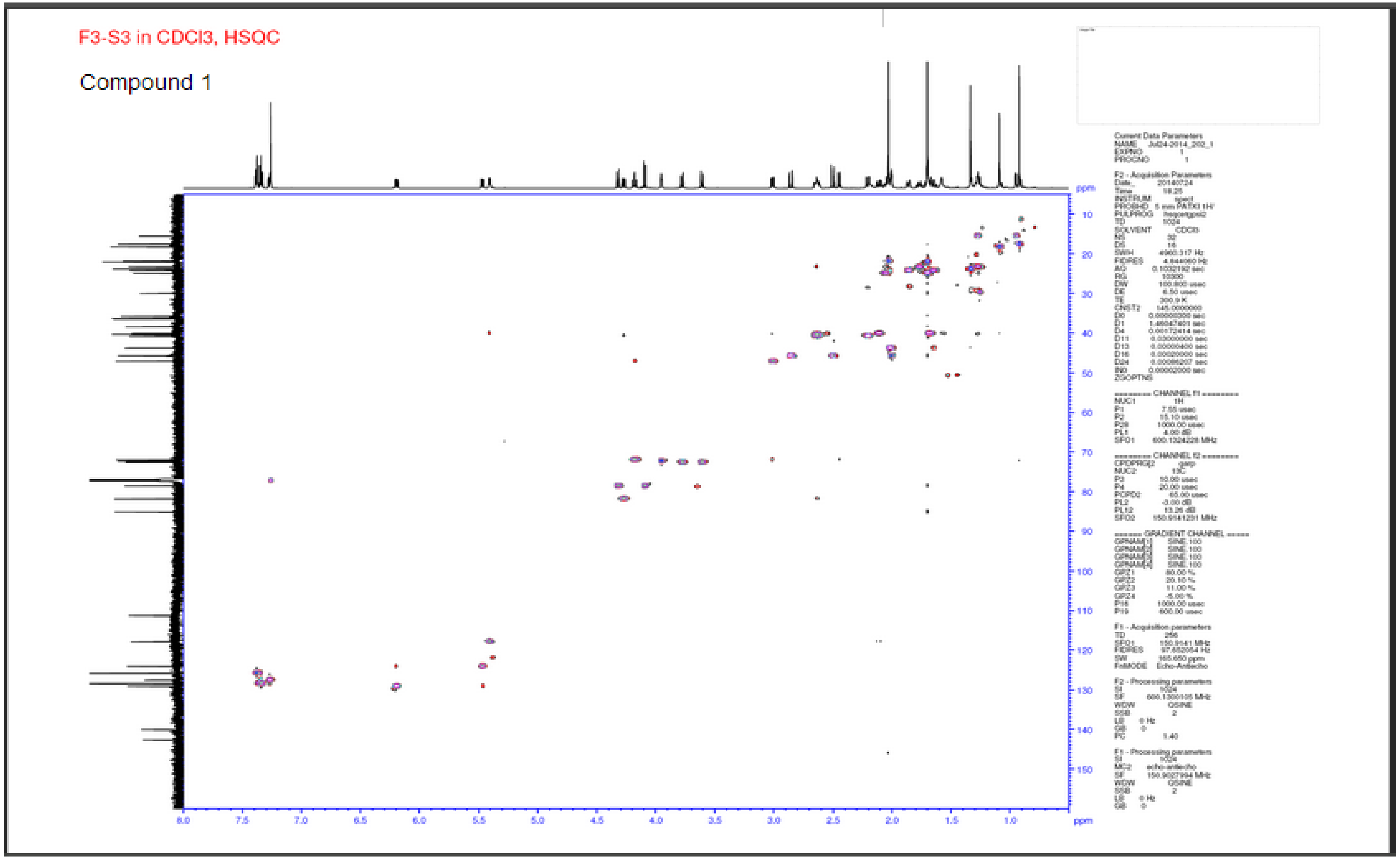

Figure S5. HSQC NMR spectrum (600MHz, $CDCl_3$, 303K) of compound 1

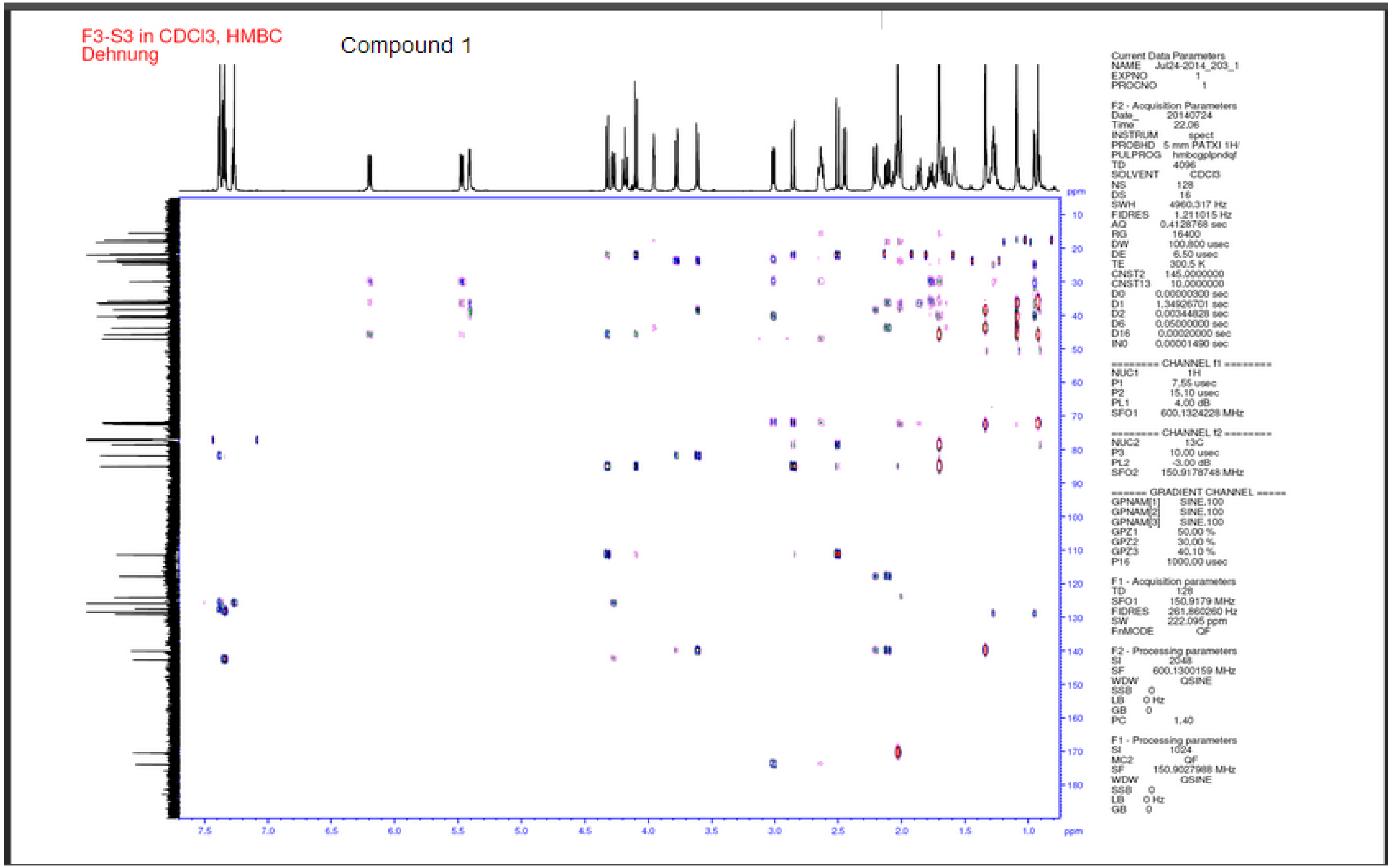

Figure S6. HMBC NMR spectrum (600MHz, $CDCl_3$, 303K) of compound 1

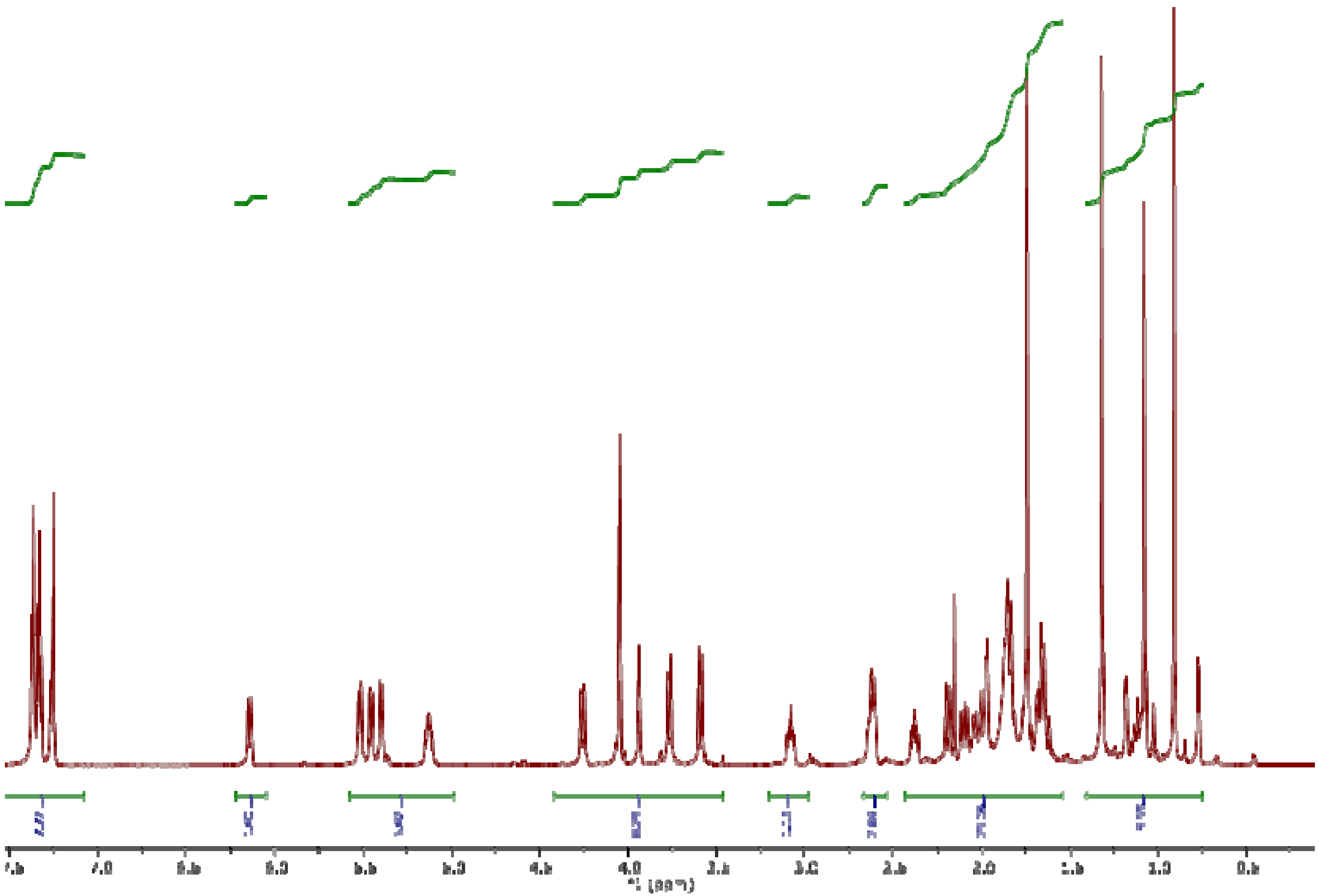

Figure S7. ^{1}H NMR spectrum (600MHz, $CDCl_3$, 303K) of compound **2**

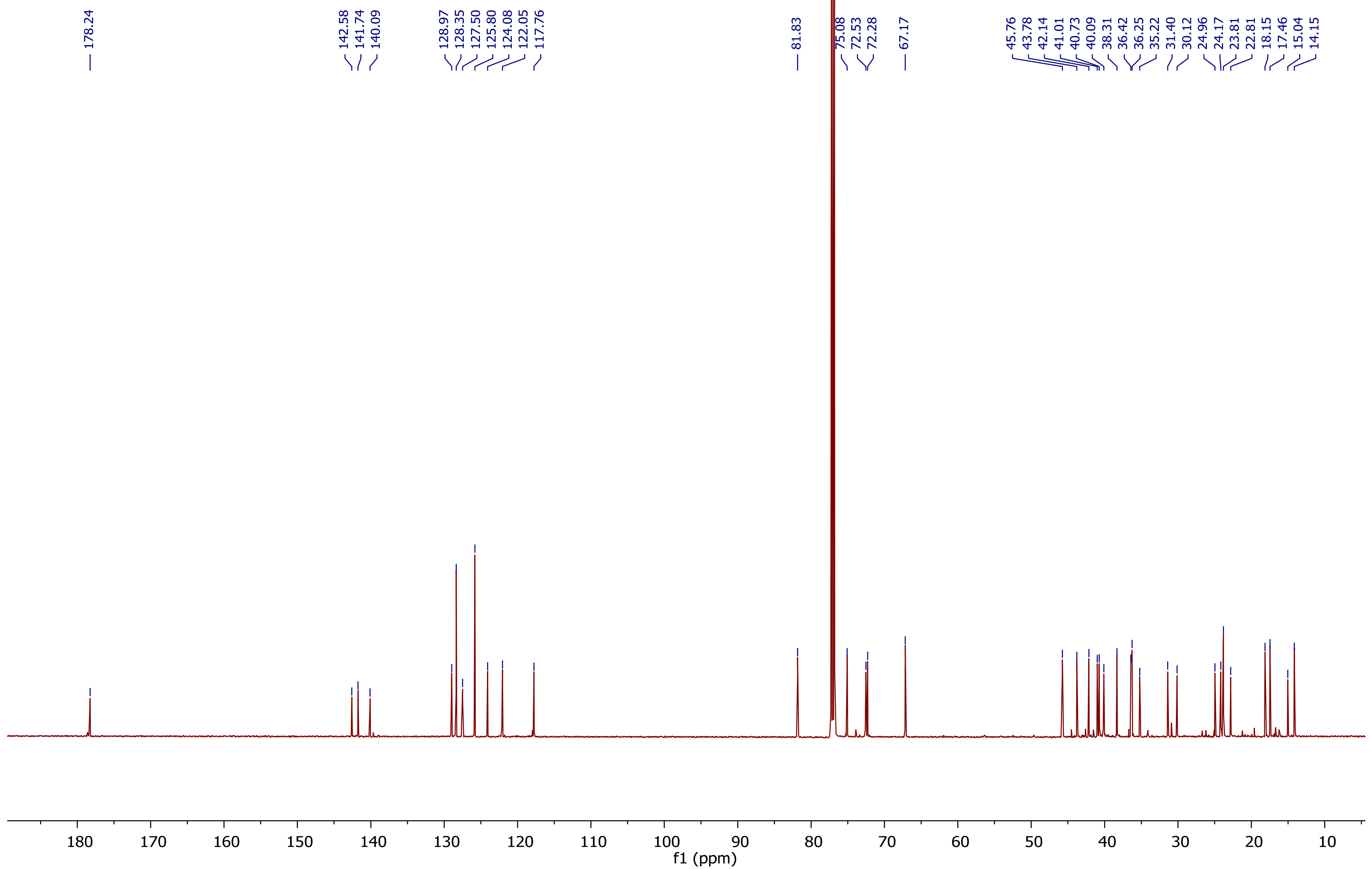

Figure S8. ^{13}C-NMR spectrum (150MHz, $CDCl_3$, 303K) of compound **2**

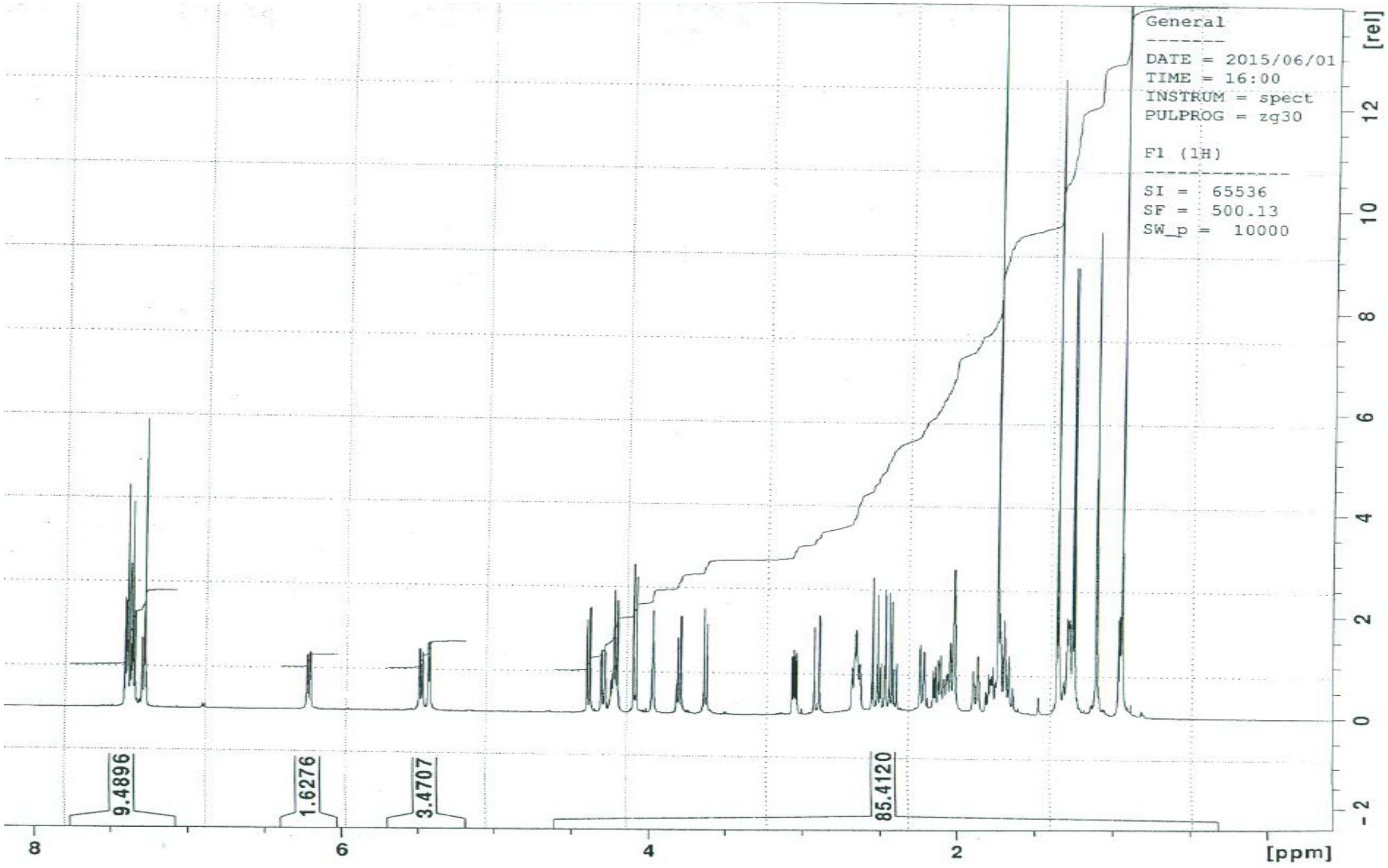

Figure S9. ^{1}H NMR spectrum (600MHz, $CDCl_3$, 303K) of compound **3**

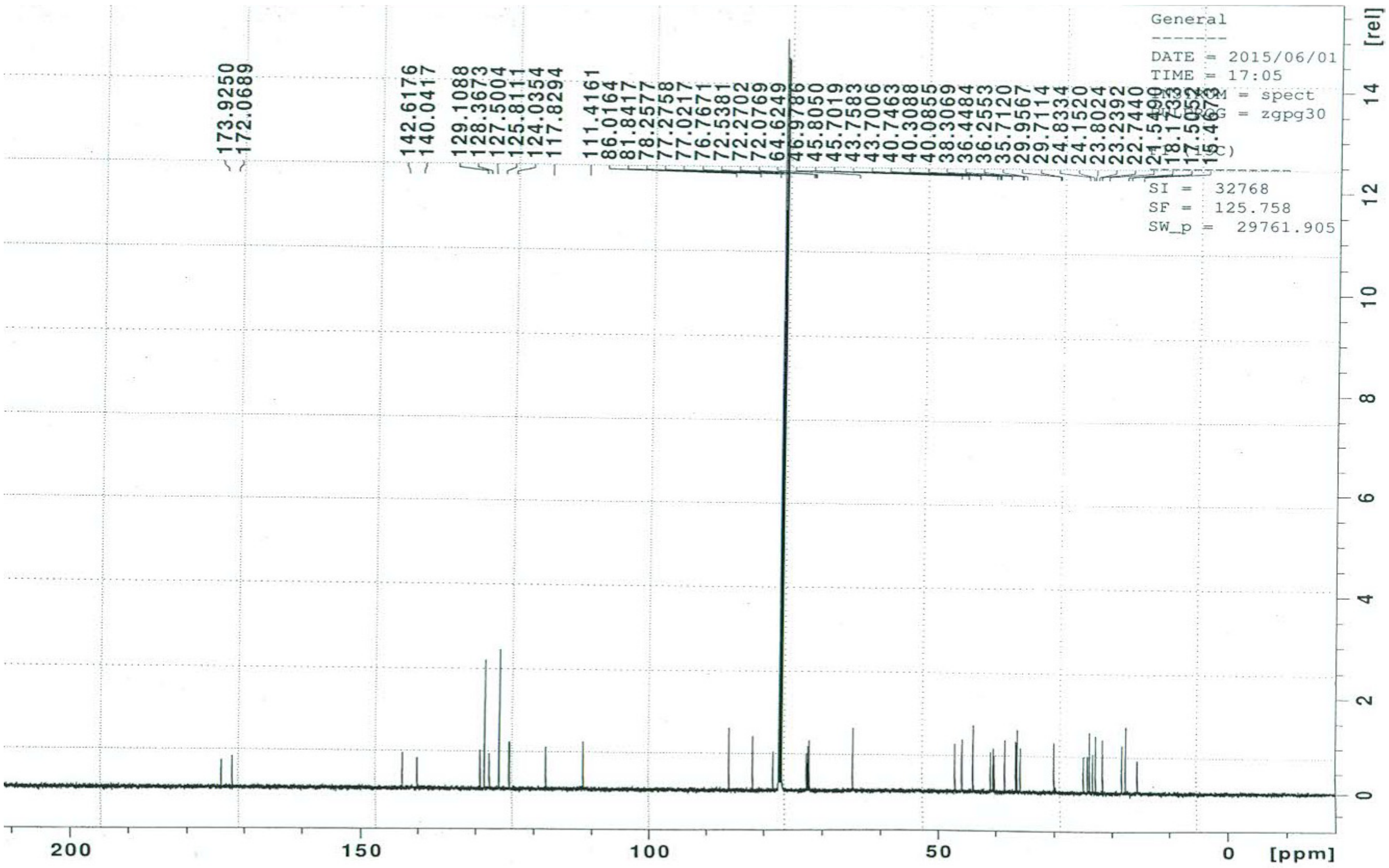

Figure S10. ^{13}C NMR spectrum (150MHz, $CDCl_3$, 303K) of compound **3**

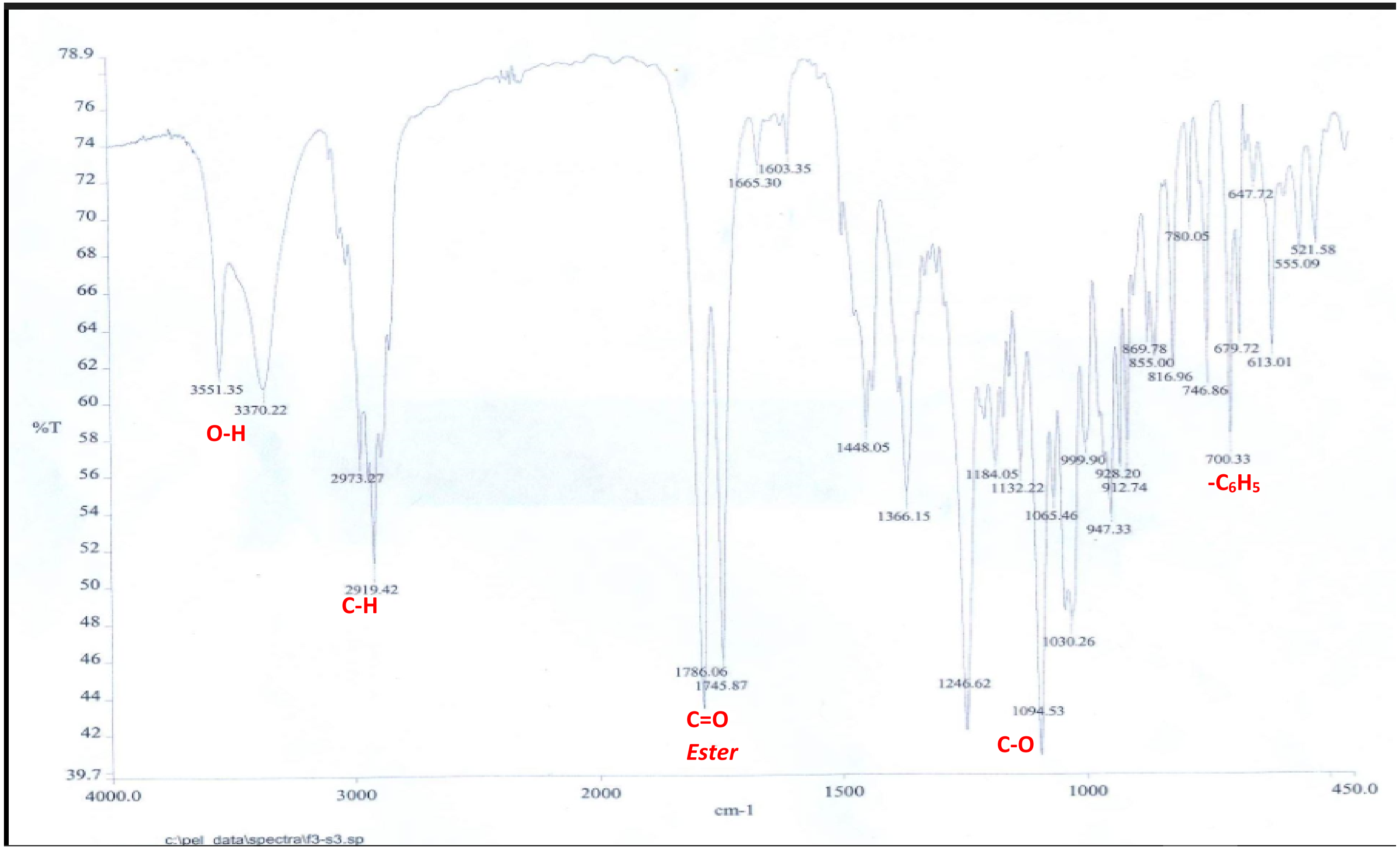

Figure S11. IR spectrum of compound **1**

Table S1. Jurkat cell viability assay data for compounds **1** - **3**

Concentration (μM)	7-hydroxy dichapetalin P (**1**)	Dichapetalin A (**2**)	Dichapetalin X (**3**)	Curcumin
	Cell viability (%)	Cell viability (%)	Cell viability (%)	Cell viability (%)
3.125	58.1	64.0	46.5	65.6
6.25	60.5	35.2	24.9	47.5
12.5	62.5	25.5	4.5	37.8
25	48.8	14.4	2.0	11.3
50	0.0	0.0	0.0	0.0

Table S2. HL-60 cell viability assay data for compounds **1** - **3**

Concentration (μM)	7-hydroxy dichapetalin P (**1**)	Dichapetalin A (**2**)	Dichapetalin X (**3**)	Curcumin
	Cell viability (%)	Cell viability (%)	Cell viability (%)	Cell viability (%)
3.125	78.4	74.8	77.5	97.9
6.25	71.3	67.4	55.0	92.5
12.5	72.4	46.0	4.0	67.9
25	59.5	16.0	1.7	25.9
50	9.2	0.0	2.2	0.0

Table S3. CEM cell viability assay data for compounds **1** - **3**

Concentration (μM)	7-hydroxy dichapetalin P (**1**)	Dichapetalin A (**2**)	Dichapetalin X (**3**)	Curcumin
	Cell viability (%)	Cell viability (%)	Cell viability (%)	Cell viability (%)
3.125	69.0	66.4	58.5	87.7
6.25	60.6	58.7	31.2	85.8
12.5	63.8	43.1	3.8	74.8
25	48.7	15.5	4.5	35.6
50	5.6	0.0	0.0	0.0
